# PLATO software provides analytic framework for investigating complexity beyond genome-wide association studies

**DOI:** 10.1038/s41467-017-00802-2

**Published:** 2017-10-27

**Authors:** Molly A. Hall, John Wallace, Anastasia Lucas, Dokyoon Kim, Anna O. Basile, Shefali S. Verma, Cathy A. McCarty, Murray H. Brilliant, Peggy L. Peissig, Terrie E. Kitchner, Anurag Verma, Sarah A. Pendergrass, Scott M. Dudek, Jason H. Moore, Marylyn D. Ritchie

**Affiliations:** 1 Institute for Biomedical Informatics, Departments of Genetics and Biostatistics and Epidemiology, Perelman School of Medicine, University of Pennsylvania, Philadelphia, PA 19104 USA; 2 Biomedical and Translational Informatics Institute, Geisinger Health System, Danville, PA 17821 USA; 3 Department of Biochemistry and Molecular Biology, Center for Systems Genomics, Eberly College of Science, The Pennsylvania State University, University Park, PA 16802 USA; 4 Essentia Institute of Rural Health, Duluth, MN 55805 USA; 5 Marshfield Clinic Research Institute, Marshfield, WI 54449 USA

## Abstract

Genome-wide, imputed, sequence, and structural data are now available for exceedingly large sample sizes. The needs for data management, handling population structure and related samples, and performing associations have largely been met. However, the infrastructure to support analyses involving complexity beyond genome-wide association studies is not standardized or centralized. We provide the PLatform for the Analysis, Translation, and Organization of large-scale data (PLATO), a software tool equipped to handle multi-omic data for hundreds of thousands of samples to explore complexity using genetic interactions, environment-wide association studies and gene–environment interactions, phenome-wide association studies, as well as copy number and rare variant analyses. Using the data from the Marshfield Personalized Medicine Research Project, a site in the electronic Medical Records and Genomics Network, we apply each feature of PLATO to type 2 diabetes and demonstrate how PLATO can be used to uncover the complex etiology of common traits.

## Abstract (web summary)

Centralized infrastructure to support analyses involving complexity beyond genome-wide association studies is broadly needed. Here, Ritchie and colleagues develop PLATO, a software tool to process and integrate various methods for this task.

## Introduction

Genome-wide association studies (GWAS) have identified thousands of SNP-phenotype associations over the past decade. However, the trend for common traits has been consistent: the majority of SNPs demonstrate a modest effect size on the trait with which they are associated. This pattern, referred to as “missing heritability”, has been widely discussed^1–3^, and GWAS is often criticized as a one-dimensional tool that does not embrace the complexity that exists in biology^1–3^. Some of the burgeoning areas of complexity beyond GWAS include rare and structural variation, the environment and gene–environment interactions, and gene–gene interactions. However, such analyses involve multiple data types and analytical tools. To date, there has been no centralized infrastructure built to handle and integrate these data and associations.

**Fig. 1:**
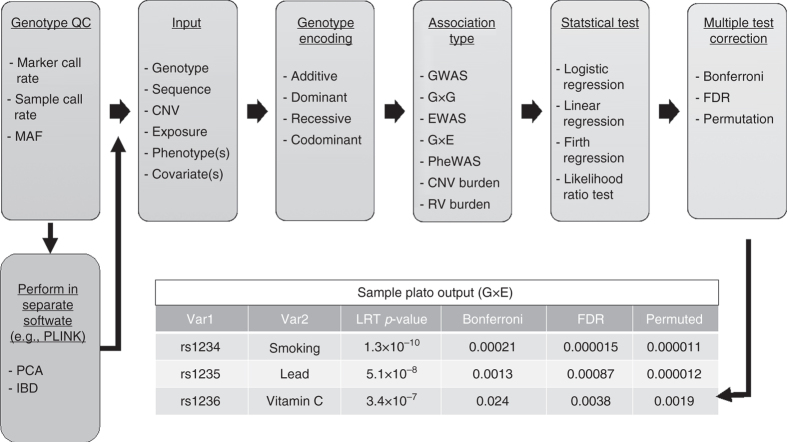
PLATO pipeline. This flow chart depicts a typical research pipeline using PLATO. Call rates and minor allele frequency (MAF) can be executed in PLATO and other options for QC are performed elsewhere. A variety of data types can be used as inputs to PLATO, including PLINK files (genotype data), VCF files (sequence data), copy number variant (CNV) data, and exposure data, as well as one or more phenotypes and covariates for adjustment. PLATO offers flexibility for genetic encoding type (additive, dominant, recessive, and codominant are available). Genome-wide association studies (GWAS), gene–gene (G×G) interactions, environment-wide association studies (EWAS), gene–environment (G×E) interactions, phenome-wide association studies (PheWAS), CNV burden analysis, and rare variant (RV) burden analysis are all available in PLATO. Statistical test options currently include logistic, linear, and Firth regression and the likelihood ration test. Finally, Bonferroni, FDR, and permutations can be used to adjust for multiple tests. PLATO output includes an extensive set of statistical results in tab delimited format for ease of use. Included in this Figure is an example of a subset of the results included in PLATO output

While quality tools for data cleaning, handling population structure and related samples, and performing GWAS and linkage studies have been established^4–6^ and a number of independent methods for complexity exist (Table 1), there is need for an integrated analytic tool to investigate genetic and environmental factors by modeling the complexity involved in the development of common traits and diseases. We offer the PLatform for the Analysis, Translation, and Organization of large-scale data (PLATO) software as a multifaceted, unified tool for investigating complexity, including genetic interactions, environment-wide association studies (EWAS)^7^ and gene–environment interactions, phenome-wide association studies (PheWAS)^8^, and copy number and rare variant analyses (Fig. 1). Use of PLATO avoids the need to develop a pipeline that potentially involves downloading several packages, converting the data for each package, learning commands for each method, and outputting the data in a readable format, each stage an opportunity for user error. Instead, PLATO involves a single download, a single set of commands, standardized input format, and a tab delimited output file with detailed statistical results. The goal of PLATO is to provide a single platform to perform methods that include standard association approaches as well as those that capture complexity. We see this as especially useful for researchers without an already established pipeline, making complex association analysis accessible to a larger set of investigators.

**Table 1:** An example of the features available in PLATO and other common genomics software

	PLATO	PLINK^4^	GCTA^59^	R/bioconductor^a^
*Association analysis*				
Genome-wide association study	X	X	X	GenABEL^60^
Environment-wide association study	X			PheWAS^61^
Phenome-wide association study	X			PheWAS
Differential CNV burden analysis	X			R
Differential gene expression	X			ArrayTools^62^
Gene set enrichment analysis				GSEA^63^
Gene×gene interaction	X	X		SNPassoc^64^
Gene×environment interaction	X	X	X	CGEN^65^
Differential rare variation analysis	X			R, podkat^66^
*Types of statistical tests*				
Logistic Regression	X	X		R
Linear Regression	X	X		R
Firth Regression	X			GWASTools^67^
Likelihood ratio test	X	X		R
Auto-detect regression type	X			GenABEL
Mixed linear model association			X	GENESIS^68^
Family-based association		X		gap^69^
Estimation of variance explained			X	R
Polygenic modeling				gap
Meta-analysis			X	gap
*Genetic encodings supported*				
Additive encoding	X	X	X	GenABEL
Dominant encoding	X	X		GenABEL
Recessive encoding	X	X		GenABEL
Codominant encoding	X	X		SNPassoc
Overdominant encoding				SNPassoc
*Multiple test correction*				
Bonferroni	X	X		R
FDR	X	X		R
Permutation	X	X		SNPassoc
*QC filtering*				
Marker call rate	X	X		SNPRelate^57^
Sample call rate	X	X		SNPRelate
MAF	X	X	X	SNPRelate
LD Pruning		X	X	SNPRelate
IBD		X	X	SNPRelate
PCA		X	X	SNPRelate

^a^Bioconductor is a repository in R that includes many packages. While there may be other packages that perform the same or similar test, we have selected one for illustrative purposes

As a use case, we apply methods implemented in PLATO to the data from the Marshfield Personalized Medicine Research Project (PMRP), a site in the electronic MEdical Records and GEnomics (eMERGE) Network. Marshfield PMRP is unique in its collection of multiple data types for thousands of samples: genotype, sequence, exposure, and copy number variant (CNV) data, as well as multiple phenotypes derived from electronic health record (EHR) data. We describe results from main effect, rare variant, CNV, gene–environment interaction, and genetic interaction analyses for type 2 diabetes (T2D). We also explore potential pleiotropy, the effect of a single locus on multiple traits, by performing a PheWAS with 16 phenotypes in the Marshfield PMRP. Results from these analyses demonstrate the need to explore multiple modes of complexity within the same data set to gain a richer understanding of the mechanisms underlying common diseases.

## Results

### PLATO replicated known signal for a SNP in *TCF7L2*

**Fig. 2:**
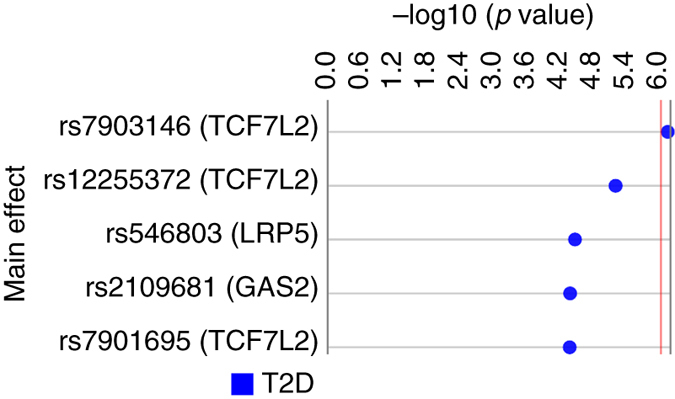
Five main effect results with uncorrected *p* value < 0.0001. Main effect analysis was performed for 33,683 SNPs and 3374 samples (835 cases, 2539 controls) using logistic regression in PLATO. This Synthesis View^58^ plot denotes the SNP (nearest gene) on the left and the track to the right displays the –log10 of the uncorrected main effect *p* value. The *red line* denotes the Bonferroni threshold for corrected significance (uncorrected *p*: 1.48 × 10^−6^; *α* = 0.05, 33,683 tests)

To demonstrate that PLATO is capable of identifying loci known to be associated with T2D, we performed main effect SNP association analysis for SNPs in genes known to relate to the disorder. We compiled this SNP list by performing a gene PubMed search with the phrase “type 2 diabetes”. This search yielded 1617 genes (Supplementary Data 1). All SNPs on our genotyping platform that fell into these genes with a 10 kb upstream and downstream gene boundary were included in our analysis (37,608 total SNPs before QC). Of the 33,683 SNPs that passed our QC criteria in this sample set, one was found to be associated with T2D with a Bonferroni corrected *p* < 0.05 (33,683 tests) (Fig. 2). This top result was for SNP rs7903146 in the gene *TCF7L2* with an uncorrected *p* value of 1.12 × 10^−6^ and a Bonferroni corrected *p* value of 0.042 (false discovery rate (FDR): 0.042). Supplementary Data 2 includes all results from the main effect analysis.

### Rare variant analysis yielded no statistically significant results

While recent technological advances have presented an opportunity for rare variant discovery, rare and low-frequency variant analysis requires special consideration. As these variants are individually uncommon, they are often statistically underpowered for detecting phenotypic association^9^. To circumvent this challenge, binning or collapsing methodologies are often utilized. Binning methods aim to aggregate multiple rare variants into a single, defined genetic variable as a means of increasing composite allele frequency and improving statistical power. BioBin^10^ is a bioinformatics tool developed specifically for the automated binning of variants into user-designated biological features using publically available biological information. BioBin performs multi-level binning of variants into biological features such as genes, pathways, protein families, regulatory regions, and evolutionary conserved regions by accessing the Library of Knowledge Integration (LOKI) data repository (further described in “Methods” section). BioBin is not built into PLATO; it is a stand-alone tool and was utilized prior to PLATO analysis.

Subjects from the Marshfield PMRP were sequenced as part of the eMERGE-PGX study^11^ using PGRNseq^12^, a next-generation, high throughput sequencing platform developed by the NIH Pharmacogenomics Research Network (PGRN) for the targeted capture of 84 pharmacogenes. We restricted our analysis to genes that overlap between the targeted PGRNseq platform and the PubMed T2D gene list (Supplementary Data 3). Our BioBin gene analysis produced 43 bins containing variants with a MAF below 0.05. Of these bins, three were found to be associated with T2D with a *p* value < 0.05 using PLATO, but none were significant when adjusting for the number of bins tested. These results are illustrated in Supplementary Fig. 1. The top result was for low frequency variants in *SLC47A1* with an uncorrected *p* value of 0.00419. Supplementary Data 4 lists all results from the rare variant analysis.

### Interaction between missense SNP in *GANC* and alcohol consumption

**Fig. 3:**
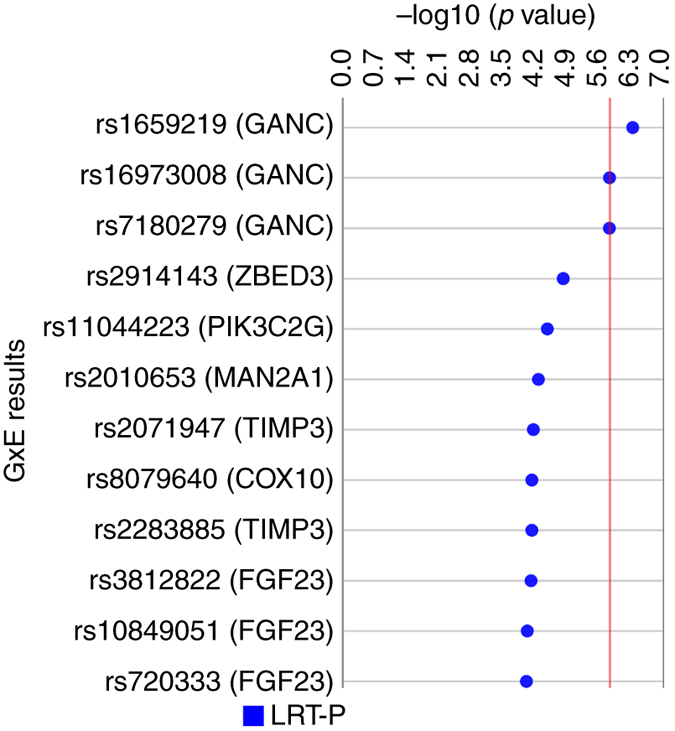
SNP-*Alcohol 30-Day Frequency* interaction results. SNP-environment analysis was performed for 33,622 SNPs and 2044 samples (390 cases, 1654 controls) using logistic regression in PLATO. This Synthesis View^58^ plot denotes the SNP (nearest gene) on the left and the track to the right displays the –log10 of the uncorrected LRT *p* value. The *red line* denotes the Bonferroni threshold for corrected significance (uncorrected LRT p: 1.49 × 10^−6^; *α* = 0.05, 33,622 tests)

In a previous T2D EWAS, we investigated 314 environmental variables for their association with T2D in the Marshfield PMRP using PLATO software^13^. For the current study, we were specifically interested in exploring gene-environment interactions using the top result from the previous EWAS: the number of days of the last 30 where one or more alcoholic beverages was consumed (*Alcohol 30-Day Frequency*). Here, we tested this exposure for interaction with the PubMed filtered loci. The results of this G×E interaction analysis yielded 12 SNPs that were found to interact with *Alcohol 30-Day Frequency* with a LRT *p* value less than 1 × 10^−4^ (Fig. 3). The result with the lowest LRT *p* value was for a missense SNP, rs1659219, in glucosidase alpha neutral C (*GANC*) with an uncorrected LRT *p* value of 4.72 × 10^−7^, Bonferroni corrected *p* value of 0.016 (33,622 tests), and FDR: 0.016. Supplementary Data 5 includes all results from the gene–environment interaction analysis.

### Alcohol consumption and CNV deletion burden

In recent years, CNVs and their genome-wide burden have been implicated in numerous diseases^14^. We assessed CNV deletion, duplication, and total burden and did not observe any significant *p* values at the *α* = 0.05 level. Supplementary Data 6 shows the results for all three CNV burden classifications.

We next looked for CNV burden-environment interactions. Here, we tested deletion, duplication, and total burden for interactions with exposure measures derived from the PhenX Toolkit and the Diet History Questionnaire (DHQ) (see Methods for description). Two CNV–environment interactions achieved an uncorrected LRT *p* value below the 0.01 threshold, though none of these results achieved Bonferroni significance at the *α* = 0.05 level. These results included: from PhenX, the interaction between *Mania: More Sexually Active* and deletion burden (uncorrected LRT *p* value = 0.00402) as well as the DHQ measure *Grams of Alcohol Consumed* and deletion burden (uncorrected LRT *p* value = 0.00793).

Given the recurrence of gene-environment interaction results involving alcohol consumption, we hypothesized that if *GANC* is important in T2D, then it is possible that deletions in *GANC* or genes involved with *GANC* could also play an important role in the condition. To test this, we mapped only genes present in the KEGG pathways that also contain *GANC*: metabolic pathways, starch and sucrose metabolism, and galactose metabolism, to CNVs. We then applied a permutation test developed for CNV annotation, the details of which are outlined previously^15^ and in Methods.

Of the 203 genes that were mapped to deletions, we found two genes with significantly higher numbers of cases with a deletion overlapping the gene than controls, after adjusting for the total number of cases and controls, with a permuted *p* value threshold of 0.05. The top result was *NDST4* (permuted *p* value = 0.010) followed by *HPSE2* (permuted *p* value = 0.012).

### Interactions between SNPs in *GAD1* and *GAD2*

Gene–gene interactions can become computationally intensive when explored as comprehensive pairwise combinations. Further, the multiple correction penalty can lead to missing true positive SNP–SNP models if no filtering approach implemented. To reduce the number of tests, we assessed two filtering strategies: main effect and knowledge-based filtering. Main effect filtering involves selecting only SNPs meeting a chosen main effect significance threshold for subsequent pairwise interactions. The knowledge-based approach limits the search to only SNP–SNP models that have an established biological relationship. We used Biofilter software^16^ to create our knowledge-based filtered SNP–SNP model list (further described in Methods).

**Fig. 4:**
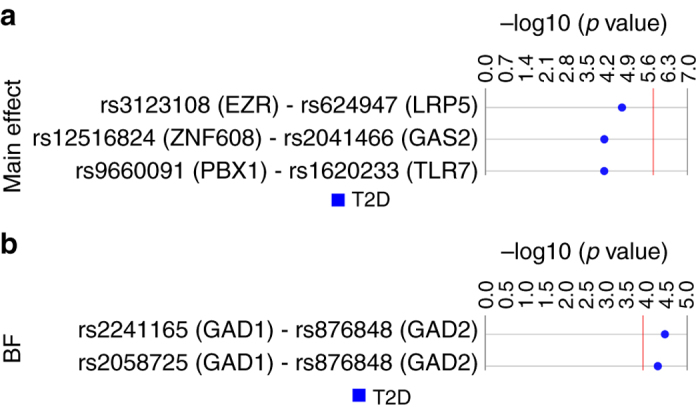
Top main effect filtered and Biofilter G×G results. **a** SNP–SNP analysis was performed for 32,640 main effect filtered models and 3374 samples (835 cases, 2539 controls) using logistic regression in PLATO. This Synthesis View^58^ plot denotes the SNPs (nearest genes) on the *left* and the track to the *right* displays the –log10 of the uncorrected LRT *p* value. The *red line* denotes the Bonferroni threshold for corrected significance (uncorrected LRT *p*: 1.53 × 10^−6^; *α* = 0.05, 32,640 tests). **b** SNP–SNP analysis was performed for 404 Biofilter models and 3374 samples (835 cases, 2539 controls) using logistic regression in PLATO. This Synthesis View^58^ plot denotes the SNPs (nearest genes) on the *left* and the track to the *right* displays the –log10 of the uncorrected LRT *p* value. The *red line* denotes the Bonferroni threshold for corrected significance (uncorrected LRT *p*: 1.24 × 10^−4^; *α* = 0.05, 404 tests)

Our main effect filtering method yielded 3 models with uncorrected LRT *p* values less than 0.0001 (Fig. 4a). No models were significant when requiring a Bonferroni correction (*α*: 0.05, 32,640 pairwise SNP–SNP models derived from the PubMed SNP list) or FDR. The top model involved SNPs rs3123108 in ezrin (*EZR*) and rs624947 in LDL receptor related protein 5 (*LRP5*) (uncorrected LRT *p* value: 1.86 × 10^−5^). Supplementary Data 7 includes all results for the main effect analysis. Biofilter created 404 SNP–SNP models using the PubMed SNP input. Of these, 2 models met a Bonferroni corrected LRT *p* value < 0.05 (Fig. 4b). The top model included SNPs rs2241165 in *GAD1* and rs876848 near *GAD2* (uncorrected LRT *p* value: 3.54 × 10^−5^; Bonferroni: 0.014; FDR: 0.011). Supplementary Data 8 includes all results from the Biofilter interaction analyses.

### Identification of eleven SNPs associated with three lipid traits

**Fig. 5:**
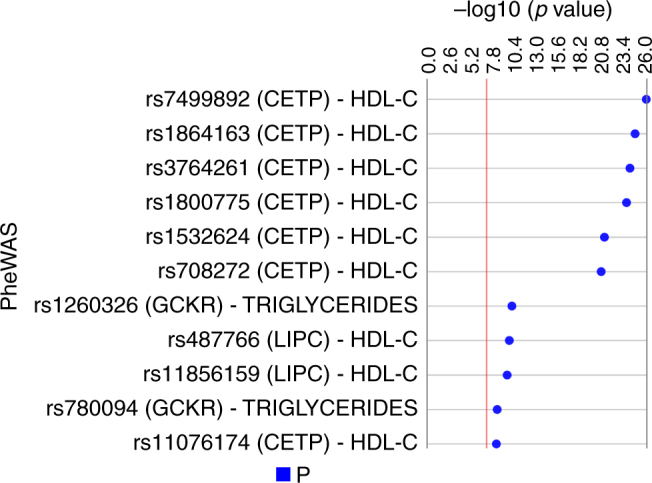
The top PheWS results. PheWAS using linear or logistic regression (depending on whether the outcome was binary or continuous) in PLATO. This Synthesis View^58^ plot denotes the SNP (nearest genes) and phenotype association on the *left* and the track to the *right* displays the –log10 of the uncorrected *p* value. The *red line* denotes the Bonferroni threshold for corrected significance (uncorrected LRT *p*: 9.30 × 10^−8^; *α* = 0.05, 547,536 tests)

To demonstrate the utility of PLATO as a tool to explore potential pleiotropy, we performed a PheWAS with 33,596 T2D PubMed SNPs that passed the QC threshold in this sample set against 16 phenotypes. 11 SNP-phenotype associations demonstrated Bonferroni corrected *p* values less than 0.05 (537,536 tests) (Fig. 5). The top result included SNP (rs7499892) in gene *CETP*, which was associated with HDL cholesterol with an uncorrected *p* value of 1.04 × 10^−26^. Supplementary Data 9 displays all PheWAS results with *p* < 0.01.

### PLATO usage

When using PLATO, it is difficult to determine a “standard” analysis due to the flexibility of the tool and the wide variety of options available to a user. However, given a sample execution, we can define how the computation will scale with a variety of parameters. We ran a standard case-control GWAS using logistic regression in PLATO for 20,000 samples and 552,293 markers, 5 commonly used covariates (sex, age, BMI, PC1 and PC2), parallelizing using 32 threads, and this took 49 min. The majority of the computational burden comes from the linear algebra routines used in regression, which scales linearly in the number of samples and quadratically in the number of variables. Thus, doubling the number of samples should double the time, and doubling the number of covariates will quadruple the time. One important note is that a categorical covariate of N levels will generate N-1 columns, so care should be taken to minimize the number and levels of the categorical covariates. Additionally, as mentioned previously, because this is a parallel problem, doubling the number of models will double the runtime, but doubling the number of computational cores will halve the runtime. The memory requirements are largely driven by the size of the genetic data, a good rule of thumb is that PLATO will require twice the size of the bed file for memory when using binary PLINK files.

## Discussion

Genome-wide association studies have demonstrated success at identifying genetic loci involved in a number of complex phenotypes. If the environment and gene–environment interactions, gene–gene interactions, and rare and structural variations are not considered, however, the yet unexplained heritability is likely to remain elusive. We presented results using PLATO, a novel, integrative tool for identifying complex associations predictive of common traits from a diverse set of data types linked to the EHR using data from the Marshfield PMRP. Marshfield PMRP is a valuable data set with genome-wide SNP, environmental exposure, CNV, and sequence data, as well as numerous phenotypes pulled from the EHR.

As a proof of concept, we first assessed PLATO’s ability to identify known T2D loci in a main effect analysis. We identified one Bonferroni significant result for a SNP in transcription factor 7-like 2 (*TCF7L2*). This SNP has previously been shown to be involved in T2D^17^. While this was not a GWAS, as only a subset of SNPs were utilized, the methodology could be applied to a genome-wide set of SNPs for any genome-wide analysis.

Our rare variant analysis did not yield any results meeting a Bonferroni corrected criteria but did reveal three genes associated with T2D (with *p* < 0.05) using BioBin software to bin the rare variants within each T2D-related gene bin. The top result was for gene solute carrier family 47 member 1 (*SLC47A1*), variants of which have demonstrated involvement in response to metformin in individuals with diabetes^18^.

In our previously published T2D EWAS^13^ the exposure, *Alcohol 30-Day Frequency*, was the top result. In this current study, we tested this previously identified exposure for interaction with PubMed T2D SNPs and found it to interact with a missense SNP in *GANC. GANC* is a glycosyl hydrolase enzyme that hydrolyzes the glycosidic bond between two or more carbohydrates. This is an important enzyme in glycogen metabolism that is associated with diabetes susceptibility, and alpha glucosidase inhibitors have been used clinically to lower glucose levels in diabetics^19^. This finding, if validated, would help to further elucidate the complex interaction between lifestyle and genetics underlying this condition. Future investigation in model organisms will allow for additional verification as well as insights into the mechanisms of this finding.

The CNV analysis further revealed the importance of investigating complex interactions with the environment. Our main effect burden analysis identified no significant results; yet, when we considered interactions of different burden types with environmental exposures, we found two results with uncorrected LRT *p* values less than 0.01. Of these results, one was for alcohol intake and deletion burden, which was notable, considering the alcohol consumption results revealed from the SNP–environment analysis. Due to this commonality, we investigated whether any genes involved with *GANC* may be enriched for deletion, and identified genes N-deacetylase/N-sulfotransferase 4 (*NDST4*) and heparanase (*HPSE2*)*. NDST4* has been found to be associated with levels of circulating resistin, a hormone reported to be associated with insulin resistance, T2D, and cardiovascular disease^20^. Another study found an association between a SNP near *NDST4* and a phenotype similar to *Alcohol 30 day frequency*: *Maximum number of alcoholic drinks consumed in a 24-h period* ^21^. A study focused on type 1 diabetes (T1D) in mouse models suggests that pancreatic islets, containing insulin-secreting β-cells, are susceptible to damage by heparanase and the inhibition of heparanase could be protective for T1D^22^. The consistency we observed in our SNP-environment and CNV-environment results demonstrate the importance of measuring exposures through multiple modes, as each alcohol measure was obtained through different questionnaires (PhenX and DHQ).

Two different filtering techniques were utilized for our genetic interaction analysis in order to reduce the number of tests performed: main effect filter and knowledge-based filter. None of the main effect filtered SNP–SNP models achieved Bonferroni significance. The top result involved SNPs in gene ezrin (*EZR*) and LDL receptor related protein 5 (*LRP5*). The knowledge-based filtering method reduced the number of tests greatly, compared to our main effect filter. Two of these models met a Bonferroni corrected threshold for significance. The two models included SNPs in genes glutamate decarboxylase 1 (*GAD1*) and glutamate decarboxylase 2 (*GAD2*), both relating to T2D, as glutamic acid decarboxylases are targets of autoantibodies involved in T2D^23^.

We also implemented a PheWAS for our T2D-related SNPs and 16 phenotypes from the Marshfield PMRP. PLATO identified 11 SNP-phenotype associations with *p* values passing the Bonferroni significance threshold when adjusting for the number of tests. These top results involved SNPs in cholesteryl ester transfer protein, plasma (*CETP*), which were associated with HDL cholesterol, and SNPs in glucokinase (hexokinase 4) regulator (*GCKR*), associated with triglycerides. *CETP* encodes a plasma protein involved in transfer of cholesteryl ester from high density lipoprotein (HDL) to other lipoproteins. The SNP in the top association result (rs7499892) in *CETP* has been found to be associated with HDL cholesterol in previous GWAS^24^. One SNP (rs1260326) in *GCKR* that we found to be associated with triglycerides was a missense SNP, which has demonstrated previous GWAS associations with triglycerides^25^, hypertriglyceridemia^26^, total cholesterol^27^, metabolite levels^28^, non-albumin^29^, waist circumference^30^, gout^31^, lipoprotein-associated phospholipase A2 activity and mass^32^, serum albumin levels^33^, serum total protein levels^33^, c-reactive protein levels^34^, 2 h glucose challenge^35^, cardiovascular disease risk factors^36^, liver enzyme levels^37^, chronic kidney disease^38^, hematological and biochemical traits^25^, urate levels^39^, and platelet count^40^. Replicating the SNP-phenotype associations described here demonstrates the capability of PLATO to identify known associations across multiple trait types.

There are some limitations to this study, which may have influenced our power. For some of the analyses (especially the rare variant and environmental analyses), the sample size was not optimal due to the cost-prohibitive nature of sequencing large sample sizes and the time required for participants to complete questionnaires. Additionally, we did not seek replication in a separate data set, as such rich, diverse, and comprehensive data types, as those collected by Marshfield PMRP, are exceedingly rare. Nevertheless, we were able to identify many results that passed the strict Bonferroni correction criteria. The Marshfield PMRP data is unique in the wide range of data types available and was, therefore, useful to showcase the capabilities and flexibility of PLATO as a tool to uncover multiple modes of complexity. Additionally, the goal of this work was to demonstrate the utility of PLATO. We do not anticipate that any of the methods described in this paper, individually, will explain the missing heritability in common traits; rather, it is through integrating these methods that we hope to understand the etiology of common traits.

We liken PLATO to a Swiss army knife for complex associations. While an entire toolbox of methods have been developed across a wide scope of domains, PLATO is a versatile and user-friendly tool for exploring several essential types of complex associations on a single platform. Many tools like PLINK, GCTA, and numerous R/Bioconductor packages have been integrated in successful workflows across the world. A major advantage to a single platform like PLATO is reduction of user time and error. For instance, there are several steps involved in establishing a workflow using multiple packages, including: (1) researching which package to utilize, (2) downloading each package (and ensuring software version is compatible with those packages), (3) gaining familiarity with different commands by reading each manual, (4) troubleshooting unique bugs and issues for each tool, (5) formatting the data for each program, and (6) outputting the data in readable format. Alternately, using PLATO involves downloading a single software; learning one set of commands; one general file format system; and a user-friendly, consistent result output file with an extensive list of statistics. Supplementary Data 10 is offered to demonstrate examples of differences between running PLATO vs. various R packages. Often for the R packages, several lines of code are required for each package, while the PLATO command involves only small argument tweaking for each type of analysis (e.g., “--interaction” when running an interaction, or “--use-trait” when using the environmental data). Further, PLATO is currently a regression-focused association methodology; yet, there are additional useful analysis strategies that can be incorporated, and PLATO is built as an open-source platform to allow add-ons from users to meet the developing needs for complex association analysis.

Genome-wide association studies have provided a foundation from which to explore the genetic components of common traits. To further explain these phenotypes, methods embracing complexity beyond GWAS are necessary. The components of PLATO described here represent a step toward modeling complexity by embracing phenotypic connections, copy number and rare variation, the exposome and gene-environment interactions, and genetic interactions. Precise, individualized medical prediction, disease prevention, and treatment are the goal in biomedical research. To achieve these goals, it is essential to integrate methods like those discussed here so as to detect genetic-environment-phenotype interrelationships. We offer PLATO as a single tool to explore diverse layers of complexity and deepen our knowledge of the nature of common diseases.

## Methods

### Marshfield PMRP and T2D case identification

The Marshfield PMRP is a biobank with ~ 20,000 subjects 18 years of age and greater who are enrolled in the Marshfield Clinic in Wisconsin^41^. Upon enrollment, volunteers complete an informed consent document with permission for continued access to the EHR, and DNA, plasma, and serum samples are collected. PMRP participants also complete questionnaires regarding smoking history, occupation, physical activity, and diet. The IRB of Marshfield Clinic approved all forms and procedures for the PMRP.

Using an algorithm developed by the eMERGE Network^42^, T2D patients were identified by their records from the Marshfield Clinic EHR. Marshfield samples were originally selected for eMERGE based on their cataract case-control status. Criteria for T2D case status were defined as having the following in their EMR: a T2D ICD-9 diagnosis billing code, information about insulin medication, abnormal glucose or HbA1c levels, or more than two diagnoses of T2D by a clinician. T2D cases with an ICD-9 code for T1D were removed from further analyses. All control subjects were required to have at least 2 clinical visits, at least one blood glucose measurement, normal blood glucose or HbA1c levels, no ICD-9 codes for T2D or any related condition, no history of being on insulin or any diabetes related medication, and no documented family history of T1D or T2D.

### Genetic data

*Genotype data*. Genotyping of DNA samples from the Marshfield Clinic was performed using the Illumina 660W-Quad array. The data were cleaned following the eMERGE quality control (QC) pipeline developed by the eMERGE Genomics Working Group^43^. This pipeline involves evaluation of sample and marker call rate, sex mismatch, duplicate and HapMap concordance, batch effects, Hardy-Weinberg equilibrium, sample relatedness, and population stratification. QC thresholds included: marker call rate > 99%, sample call rate > 99%, and minor allele frequency (MAF) > 5%. To restrict our analysis to SNPs likely to be involved in T2D, we performed a gene PubMed search with the phrase “type 2 diabetes”, yielding 1617 genes (Supplementary Data 1) and 37,608 total SNPs before QC (that fell within a 10 kb upstream and downstream gene boundary).

*Sequence data*. Subjects from the Marshfield PMRP were sequenced as part of the eMERGE-PGX study^11, 44^ using PGRNseq^12^ capture with the Illumina HiSeq 2000. PGRNseq is a next-generation, high throughput sequencing platform developed by the NIH PGRN for the targeted capture of 84 pharmacogenes. The captured sequence includes 2 kb upstream of the selected coding regions. The 84 pharmacogenes were collaboratively chosen by PGRN and are known to have associations with drug phenotypes^12^. Further, many of these genes have been identified as actionable by the Clinical Pharmacogenetics Implementation Consortium (CPIC). The PGRNseq platform has shown 99.8% genotype concordance with orthogonal data sets from HapMap and 1000 Genomes projects^12^.

*Copy number variation*. CNVs were called from the Illumina 660W-Quad array using PennCNV software^45^. PennCNV output includes the start and stop positions and copy number state, either deletion or duplication, for each CNV region called. Both sample-wise and call-wise quality control thresholds were applied in PennCNV. Samples passed QC if they had a waviness factor between −0.0002 and 0.0002, call rate > 0.9918, log R ratio standard deviation < 0.3, and less than 600 distinct CNV events. Additionally, PCA outliers and related samples based on a pi hat > 0.3 in PLINK were removed. Individual CNV calls were included if they contained >  = 10 SNPs and had a length >  = 1 kb. Thresholds were chosen based on results from Pinto et al. (2012)^46^, which demonstrated that the majority of the CNVs called from the Illumina 660W-Quad Array were found to be in the 1–10 kb range. Therefore, choosing a more stringent threshold for length, such as 50 kb, would have resulted in the loss of a large percentage of our data.

### Exposure data

*Phenotype and Exposures* (*PhenX*) *Toolkit*. The PhenX Toolkit (www.phenx toolkit.org) was used to develop a self-administered questionnaire to assess environmental and lifestyle factors. Marshfield PMRP implemented PhenX as part of a supplement to the eMERGE project^47, 48^. Many of the PhenX measures were originally chosen to identify gene/environment associations with a primary disease of interest for PMRP: age-related cataract (smoking, alcohol, ultraviolet light exposure). Other measures were selected to validate prior PMRP questionnaire data and medical history information (demographics, physical activity, family history of heart attack, history of stroke) and the remaining measures were chosen because of the potential for future research and cross-site collaborations (hypomania/mania symptoms, hand dominance) with other sites, funded through administrative supplements to collect PhenX measures. Depending on how many questions were skipped, the time to complete the 32-page questionnaire ranged from 20 to 40 min in pre-testing. The questionnaire was mailed to all eligible subjects with a cover letter and return address envelope, a second questionnaire was mailed to increase the response rate, and subjects were offered $10 for their time. Questions included a variety of measures from the following classes: demographics, smoking, alcohol use, mania, depression, residential environment, activity, and UV exposure.

*Diet History Questionnaire*. Food frequency questionnaires (FFQs) are more demonstrative of usual intake and less expensive to implement than other tools, including weighed food records and 24-h dietary recalls as they are typically self-administered, and thus, are commonly used to assess dietary intake in epidemiologic studies. Self-administered food frequency questionnaires (FFQ) are available for ~2/3 of the PMRP samples. The DHQ (http://riskfactor.cancer.gov/DHQ/), was developed by researchers at the National Cancer Institute (NCI) and has demonstrated superiority over other commonly used Willett FFQ and similar to the Block FFQ in estimating absolute nutrient intakes^49^. All three of these FFQs produce similar results after statistical adjustment for total energy intake. The list of foods and portion sizes on the DHQ were developed from the USDA’s 1994–1996 Continuing Survey of Food Intakes by Individuals, and thus, is most appropriate for use with this study population. The DHQ includes 124 individual food items and asks about portion sizes for most foods. Additionally, there are 10 questions about nutrient supplement intake. Printing and scanning of the DHQ was done by National Computer Systems. After scanning, the data were stored in ASCII format and uploaded into the nutrient analysis software package. Diet*Calc software, available from the National Institutes of Health, was employed for the nutrient analyses of the DHQ data (http://riskfactor.cancer.gov/DHQ/dietcalc/). The DHQ is mailed to participants with appointment reminders so they can complete it prior to their appointment to save them time. Fifty-six measures of dietary intake were assessed for these exposure analyses that covered the following domains: vitamin, protein, carbohydrate, fiber, fat, cholesterol, caloric, vegetable, grain, caffeine, and alcohol intake.

### PLATO analysis

*PLATO usage*. PLATO is a command-line tool written in C++ for Linux. It was designed to adapt to a wide range of possible analyses presented by users, with emphasis on flexibility and extensibility of the tool. The example analyses presented are intended as a small sample of the capabilities of PLATO, as an exhaustive listing of all potential capabilities would not be possible in one paper. All analyses in PLATO consist of a sequence of pipelined steps specified by the user. Typically, one will begin by loading the data, performing some routine filtering and QC steps, then running a statistical analysis, and optionally outputing the data after QC. The PLATO software can be found at: http://ritchielab.com/software/plato-download and the PLATO manual can be found at http://ritchielab.com/files/RL_software/plato-manual-2.1.pdf. All analyses in this paper were performed using PLATO version 2.1.

PLATO’s main statistical analysis that has been implemented to date is regression, both ordinary least squares and logistic. The regression models within PLATO are designed to accommodate a wide variety of analyses, from GWAS, EWAS, PheWAS, and evaluating interactions. The regression tests a set of models, which contain one or more variables of interest, typically SNPs or their interaction terms, along with covariates.

In addition to the parametric *p* values returned by the regression models, PLATO also provides the option to use permutation testing with a user-provided number of permutations. When performing a permutation, the resulting *p* value is defined to be the proportion of permutations that have a *p* value that is smaller (more significant) than the unpermuted model. For more information about permutation options, see the PLATO manual: http://ritchielab.com/files/RL_software/plato-manual-2.1.pdf.

Because the genetic data are growing at incredible rates, tools must adapt to the increased data by employing various parallelization techniques. Fortunately, the problem of running millions of models is a parallel problem and scales almost perfectly by adding more compute cores. PLATO uses both threading and message passing interface (MPI) to parallelize the regression. The use of threads works very well to parallelize, but there is a limit of the number of cores on a single machine; MPI parallelization works to scale beyond the limits of a single computer and can be used with a high-performance computing cluster, but it comes with an increased communication overhead.

*SNP main effect*. Logistic regression was used to determine main effect association for each SNP (33,683 SNPs after QC in this sample set) with T2D, adjusting for year of birth, sex, BMI, and the first three principal components (PCs), assuming an additive genetic model. 3374 samples were available for the main effect genetic analysis (835 cases, 2539 controls). While this analysis was not a genome-wide association study, per se, as it did not include genome-wide loci, the methods employed would be easily applied to any genome-wide data set for a GWAS.

PLATO Command: *load-data recode-alleles --auto load-trait logistic ---covariates --correction*

*Rare variant analysis*. To overcome the challenges of analyzing low-frequency variants, we used BioBin software as a step before PLATO analysis. BioBin aggregates variants using a flexible and biologically informed binning strategy by consulting an internal data repository called the LOKI. LOKI contains multiple databases from the public domain, including NCBI gene Entrez^50^, Kyoto Encyclopedia of Genes and Genomes (KEGG)^51^, Reactome^52^, Gene Ontology (GO)^53^, Protein families database (Pfam)^54^, and others. LOKI integrates these disparate sources and provides a comprehensive biological knowledge platform for the biologically driven binning of variants in BioBin^16^. BioBin is open-source and available for download at http://www.ritchielab.com/software/biobin-download.

BioBin is not built into PLATO and is essentially a pre-processing step where PLATO is concerned. BioBin, or other bioinformatics approaches, can be used to create the bins to pass into PLATO for a regression analysis. We will note that while PLATO will accept a VCF file as input for analysis, we chose to bin variants for reasons of power challenges when handling rare variants (further described in Results). A BioBin-Regression analysis was performed using PLATO to determine rare variant main effect association for T2D. We restricted our analysis to genes that overlap between the targeted PGRNseq platform and the PubMed T2D gene list (Supplementary Data 2). A total of 43 genes were included in this analysis. BioBin was used to perform a gene binning analysis on the selected pharmacogenes using a minor allele frequency (MAF) threshold of 0.05. Binned variants were weighted inversely proportional to their MAF using Madsen and Browning weighting^55^. To perform burden analysis, logistic regression was run using PLATO, with output from BioBin, and adjusting for sex, year of birth and BMI and model test statistics was calculated. A total of 700 European American subjects, 102 cases and 598 controls, and were used in this rare variant analysis.

PLATO Command: *load-trait --no-fid logistic ---covariates --correction*

*Environment and SNP–environment interactions*. In a previous EWAS, we investigated 314 environmental variables for their association with T2D in the Marshfield PMRP using PLATO software^13^. For the current G×E analysis, we tested the PhenX Toolkit exposure *Alcohol 30-Day Frequency* for interaction with the PubMed filtered loci (33,622 SNPs after QC in this data set) in samples for whom both genotype and PhenX data were available (2044 samples, 390 cases, 1654 controls) using logistic regression and adjusting for year of birth, sex, BMI, and the first three PCs and assuming an additive genetic model. To determine the significance of the interaction term, we performed a LRT between the full (Y = β_0_ + β_1_SNP + β_2_Exposure + β_3_SNP × Exposure) and reduced (Y = β_0_ + β_1_SNP + β_2_Exposure) models.

PLATO Command: *load-data recode-alleles --auto load-trait logistic --interaction --use-traits ---covariates --correction*

*CNV burden main effect*. CNV burden for deletion and duplication was calculated by summing the total number of base pairs in all of the deletion and duplication regions called by PennCNV separately for each sample. Total CNV burden was calculated as the sum of all of the base pairs in each CNV region per sample regardless of copy number state. The contribution of one copy and two copy CNVs to burden was treated equally. To test if CNV burden was associated with the T2D phenotype, we ran logistic regression models for each of three burden types in PLATO. The covariates included were BMI, age, sex, and the first three PCs for a total sample size of 3195, (789 cases and 2406 controls).

PLATO Command: *load-trait --dummy-samples* *logistic --models --exclude-markers --covariates --correction*

*CNV–environment interactions*. As a follow up to our CNV burden main effect analysis, we wanted to explore the possibility of interactions between CNV burden and environmental exposures, particularly to see if the results from the SNP-environment analyses were similar. Using the same methods as in the SNP–environment interactions, we performed the LRT between the full (Y = β_0_ + β_1_CNV burden + β_2_Exposure + β_3_CNV burden × Exposure) and reduced (Y = β_0_ + β_1_CNV burden + β_2_Exposure) models. We ran these logistic regression models in PLATO for all of the DHQ and continuous and binary PhenX environment variables against all three classifications of CNV burden, again adjusting for BMI, age, sex, and the first three PCs. The DHQ data set contained 445 cases and 1639 controls for a total of 2084 samples while the PhenX data set consisted of 352 cases and 1502 controls for a total sample size of 1854. Environment variables were included in the analysis if they had a sample size >  = 200. A total of 120 environment variables and three CNV burden classifications resulted in 360 total models. Additionally, the case/control distributions of each variable involved in statistically significant (uncorrected LRT *p* value < 0.01) interactions were checked post hoc to make sure there were at least 200 cases and 200 controls for each exposure measure.

PLATO Command: *load-trait --dummy-samples* *logistic --models --exclude-markers --interactions --covariates --correction*

To assess whether there was deletion enrichment in genes relating to *GANC*, we applied a permutation test using R version 3.2.1 that was previously developed for CNV annotation, the details of which are outlined previously^15^. This method tests the hypothesis that a specific gene contains more deletions in T2D cases than in controls. We first mapped the CNV start and stop positions to genes and pathways using information from KEGG in the aforementioned LOKI data repository. A CNV was mapped to a gene if it had a  >  = 1 base pair overlap with the gene. Genes from the KEGG pathways, were chosen for the analysis if they had at least 10 cases and 10 controls with a deletion. In general, the permutation test involves creating a binary matrix, with one sample per row and one gene per column, with the last column being a binary phenotype column. If a sample has a deletion mapped to the gene in the selected column, the cell is given a count of 1; if it does not, it receives a count of 0. This is performed for each gene and sample in the matrix. If a sample had more than one CNV in a given gene, it was still only given a count of 1 since our question is “Is this gene affected by a CNV?” instead of “How much of an effect does this CNV have on the gene?”. One such matrix was created for each of the three CNV classifications. From each matrix, we calculate a knowledge base (KB) score for each gene based on a normalized ratio, cases with a CNV divided by the total number of cases over controls with a CNV divided by the total number of controls. We then permute the phenotype column 10,000 times, calculating a KB score for each gene in each permutation. By ranking the original KB score and each of the permuted KB scores, we see if the original KB score is greater than it would be by chance. The *p* value is calculated by dividing the rank of the original KB score by the total number of KB scores, where the largest KB score would receive a rank of 1 and the lowest a rank of 10,001. If the original KB score is tied for rank, it is considered to be “smaller” than the matching permuted KB scores and given a lower rank. We did not apply a correction for multiple testing, such as FDR or Bonferroni, in this case because the lower boundary on the *p* value (0.000099) would make it such that reaching statistical significance would become mathematically impossible with an increasing number of tests.

*Gene–gene interactions*. PLATO has the capability to perform both comprehensive pairwise interactions, and it can run interactions on specific SNP–SNP models, if desired, by providing a model list. For this analysis, we used the SNPs from the PubMed-identified T2D genes (33,683 SNPs after QC in this data set) and LD pruned our SNPs to further filter redundant signal using an *r* ^2^ threshold of 0.7 (resulting in 22,380 SNPs).

For the main effect filter, we used an uncorrected main effect *p* value cutoff of 0.01 from the main effect analysis described previously, which left us with 256 SNPs. PLATO automatically performed pairwise interactions between every combination of these SNPs.

Biofilter software^16^ was employed as an automated knowledge-driven filter, using the data across many biological databases. Biofilter accesses several publicly available biological knowledge databases through the previously described external database compiler called the LOKI. For more information see: http://ritchielab.com/ritchielab/software/. Using Biofilter, we built 404 biologically linked SNP–SNP models from the LD pruned PubMed list. If five or more sources in LOKI demonstrated a connection between two genes in the PubMed list, all SNP combinations between the genes were generated for the model list. Providing PLATO with this list of Biofilter-generated SNP–SNP models, the analysis was restricted to only these models.

Pairwise SNP–SNP models of main effect filtered SNPs (32,640 models) as well as the Biofilter models (404 models) were all assessed using logistic regression with PLATO, assuming an additive genetic model and adjusting for year of birth, sex, BMI, and the first three PCs in 3374 samples (835 cases, 2539 controls). To determine the significance of the interaction term, we performed the LRT between the full (Y = β_0_ + β_1_SNP1 + β_2_SNP2 + β_3_SNP1 × SNP2) and reduced (Y = β_0_ + β_1_SNP1 + β_2_SNP2) models.

PLATO Commands:

Main Effect Filter: *load-data recode-alleles --auto load-trait logistic --interaction --pairwise ---covariates --correction*

Biofilter Filtered: *load-data recode-alleles --auto load-trait logistic --interaction --model --covariates --correction*

*Phenome-wide association study* (*PheWAS*). To demonstrate the utility of PLATO with simultaneous phenotype investigation, we performed a PheWAS using 16 phenotypes for each of the SNPs in the PubMed-identified T2D genes (33,569 SNPs after QC in this sample set). Phenotypes included: age-related macular degeneration (AMD), benign prostatic hyperplasia (BPH), age-related cataract, colon polyps, diverticulosis, gastroesophageal reflux disease (GERD), glaucoma, heart failure, hypothyroidism, ocular hypertension (OHT), mace on statins, venous thromboembolism (VTE), zoster, LDL cholesterol, HDL cholesterol, and triglycerides. For each SNP-phenotype pair tested, logistic or linear regression was used, depending on the whether the phenotype was binary or continuous, assuming the additive genetic model, and adjusting for year of birth, sex, BMI, and the first three PCs.

PLATO commands: *load-data recode-alleles --auto load-trait regress-auto --phewas --covariates --correction*

### Other functions of PLATO

*Filtration*. Quality control is an important step before running any analysis. PLATO is also equipped to perform major quality control steps to filter the data. The following filters can be applied to the data sets:

- Minor allele frequency: the “filter-maf” command can be used to filter all variants below the minor allele frequency threshold provided by user.
- Marker and sample missing rate: “filter-marker-call” and “filter-sample-call” can be used to filter markers and samples, respectively, according to the missing call rate thresholds provided.
- Trait missing: “filter-trait-missing” can be used to drop all samples that have a missing phenotype value.

*Concordance check*. Checking for concordance among the two data sets is also an important quality control step, especially if samples are genotyped by two platforms, sequenced separately or imputed. PLATO’s “concordance” command is used to check for similarities and dissimilarities between the two data sets. This option outputs the following type of discordances among the two data sets:

- Sample mismatch: For each sample, the summary of discordance is reported.
- Marker mismatch: For each variant, the summary of discordance across all samples is reported.
- Discordant calls: Detailed explanation of each discordant call in the two data sets.

*Preparing the data for other tools*. PLATO also allows for converting PED/MAP or BED/BIM/FAM files to other file formats such as Beagle^56^ format, Eigenstrat^57^ format and transposed PLINK^4^ format. The data can be filtered using filtration commands as described above and converted to different formats at the same time.

*VCF files*. In addition to PLINK formatted files, PLATO is also able to load VCF files. A user can provide both compressed and uncompressed VCF files, as well as specify if the file is poly-or bi-allelic. Additionally, PLATO can be used to perform a genotype concordance check between a VCF file and PLINK formatted ped and map files.

### Code availability

PLATO is open-source and freely available to academics and non-profits at http://ritchielab.com/software/plato-download.

### Data availability

The Marshfield PMRP data are publicly available at dbGaP. The SNP genotype data, diabetes case/control status, and PhenX variables are deposited in study phs000170.v2.p1. The sequence data from eMERGE-PGx is in the process of being deposited in dbGaP. The CNV data are available from the authors upon request.

## Electronic Supplementary Material

Supplementary Information

Description of Additional Supplementary Files

Supplementary Data 1

Supplementary Data 2

Supplementary Data 3

Supplementary Data 4

Supplementary Data 5

Supplementary Data 6

Supplementary Data 7

Supplementary Data 8

Supplementary Data 9

Supplementary Data 10
