# Supplementary material for: PLATO software provides analytic framework for investigating complexity beyond genome-wide association studies
Source: Nat Commun. 2017 Oct 27;8:1167. doi: 10.1038/s41467-017-00802-2 (PMC5660079; doi:10.1038/s41467-017-00802-2)
Supplement: Supplementary file 1 — Supplementary Information [file 41467_2017_802_MOESM1_ESM.pdf]

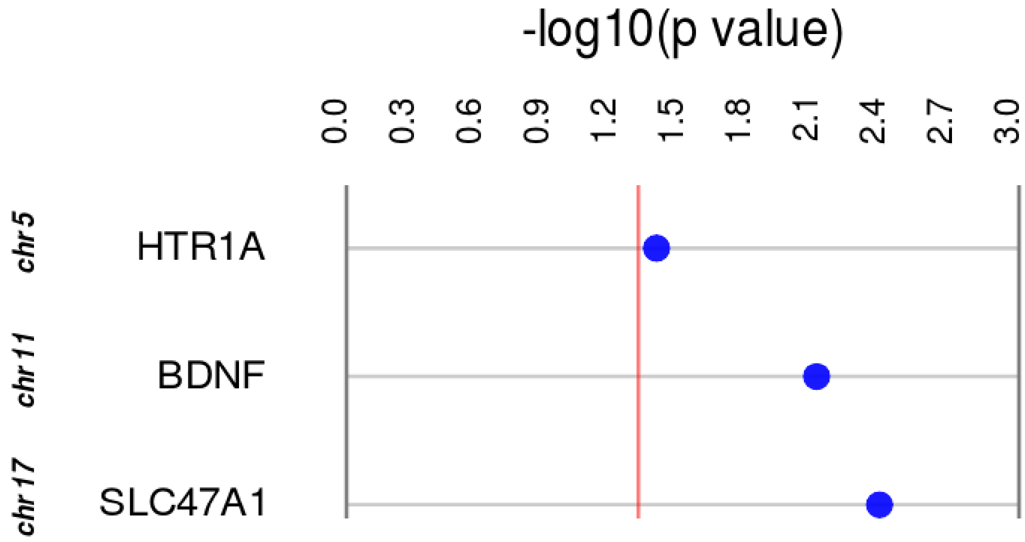

Supplementary Figure 1. Rare variant analysis was performed using BioBin[23] as a means to collapse the rare variants for 43 genes. 102 cases and 598 controls were included in this analysis. Synthesis View[21] was used to plot the –log10 of the uncorrected main effect p-value for the top 3 results that passed an uncorrected significance threshold of $p < 0.05$ (denoted by the vertical red line).
