# Supplementary material for: PLATO software provides analytic framework for investigating complexity beyond genome-wide association studies
Source: Nat Commun. 2017 Oct 27;8:1167. doi: 10.1038/s41467-017-00802-2 (PMC5660079; doi:10.1038/s41467-017-00802-2)
Supplement: Supplementary file 2 — Description of Additional Supplementary Files [file 41467_2017_802_MOESM2_ESM.pdf]

Description of Additional Supplementary Files

File Name: Supplementary Data 1
Description: a list of all genes pulled from the PubMed gene search for “type 2 diabetes”

File Name: Supplementary Data 2
Description: a complete list of the results from the SNP main effect analysis using the PubMed T2D genes

File Name: Supplementary Data 3
Description: a list of the genes included in the rare variant analysis

File Name: Supplementary Data 4
Description: a complete list of the results from the rare variant analysis

File Name: Supplementary Data 5
Description: a complete list of the results from the gene-environment interaction analysis

File Name: Supplementary Data 6
Description: a complete list of the results from the main effect copy number variant burden analysis

File Name: Supplementary Data 7
Description: a complete list of the results from the main effect filtered gene-gene interaction analysis

File Name: Supplementary Data 8
Description: a complete list of the results from the Biofilter filtered gene-gene interaction analysis

File Name: Supplementary Data 9
Description: a complete list of the results from the phenome-wide association analysis

File Name: Supplementary Data 10
Description: the commands needed to run PLATO, PLINK and select Bioconductor packages for the different analyses performed for this paper
